# ‘The Difficulty of Diagnosis Compromises Patient Care for People With Endometriosis’: Interview Study With Aotearoa New Zealand General Practitioners

**DOI:** 10.1111/hex.70390

**Published:** 2025-08-19

**Authors:** Katherine Ellis, Alina Meador, Anna P. Ponnampalam, Rachael Wood

**Affiliations:** ^1^ Department of Chemical and Process Engineering University of Canterbury Christchurch New Zealand; ^2^ Endometriosis New Zealand Christchurch New Zealand; ^3^ School of Population Health, Faculty of Medical and Health Sciences The University of Auckland Auckland New Zealand; ^4^ Department of Physiology, Faculty of Medical and Health Sciences University of Auckland Auckland New Zealand; ^5^ Pūtahi Manawa‐Healthy Hearts for Aotearoa New Zealand, Centre of Research Excellence New Zealand; ^6^ Biomolecular Interaction Centre Christchurch New Zealand

**Keywords:** chronic illness, endometriosis, healthcare access, medical practitioners, public health

## Abstract

**Background:**

General practitioners (GPs) are gatekeepers to specialist attention for endometriosis in the public system in Aotearoa New Zealand (Aotearoa‐NZ). Their perspective of the endometriosis care landscape in Aotearoa‐NZ has not been previously assessed.

**Design:**

Semi‐structured interviews.

**Participants:**

Nine GPs self‐selected to participate in interviews that were 13–28 min in duration to discuss their perspectives on endometriosis care.

**Objective:**

These interviews included their views on existing guidelines, common characteristics of endometriosis patients, and what the future of endometriosis care could look like.

**Results and Discussion:**

The GPs of this study expressed that the overloaded nature of specialist appointments in the public health system compromised the care of endometriosis patients. Resultingly, patients were subject to challenging journeys through diagnosis and management exacerbated by these health system factors. Furthermore, GPs identified that there were challenges that could prevent patients from presenting to the clinic to share their health concerns, such as a lack of awareness of ‘normal’ and pathologic menstrual‐related symptoms. Once in the clinic, patients can struggle to feel they can talk openly with their GP, due to issues such as cultural taboos around discussing menstruation. The GPs highlighted that necessary avenues to improve endometriosis care require investment into the public health system to improve its function. They also highlighted potential solutions to improve care that can be incorporated into the existing system, including expanded endometriosis guidelines, improved relationship‐building and introducing endometriosis specialist GPs or nurses.

**Patient or Public Contribution:**

Two of the authors who designed the study approach, interpreted the data and prepared the manuscript were assessed and treated for endometriosis symptoms.

## 1 Introduction

### 1.1 Endometriosis

Endometriosis, thought to affect 10% of women and people presumed female at birth, is a condition characterised by heterogeneity, diagnostic difficulty, and a wide variety of symptoms that can negatively impact all aspects of quality of life [1, 2, 3, 4]. Endometriosis is defined as the presence of endometrial‐like epithelium and/or stroma outside of the uterine cavity [5]. The pathogenesis is debated and unconfirmed, with a variety of mechanisms proposed to explain the origin of the condition affecting approximately 190 million patients worldwide [6, 7, 8, 9]. The heterogeneity in symptoms, presentation, extent and imaging effectiveness makes the diagnosis and treatment of endometriosis challenging [10, 11, 12].

### 1.2 General Practitioners in Endometriosis Care

General practitioners (GPs) can clinically diagnose suspected endometriosis in the absence of confirmatory radiological or surgical assessment [13]. They can then focus on nonsurgical treatment modalities for symptom management in the absence of confirming the presence or absence of lesions [2]. This is complicated by the identified proportion of patients who will have all the symptoms of endometriosis but do not have endometriosis lesions identified during laparoscopy [14]. However, these two patient populations both experience significant negative impacts on diverse aspects of life such as work, social interactions, romantic relationships and education [15, 16], with similar pain levels [17].

### 1.3 The Aotearoa‐NZ Context

In Aotearoa‐NZ, GPs act as the ‘gatekeepers’ to secondary care, as patients need a referral from their GP if they want to utilise the public health system and be treated by specialists. These specialists can open up different avenues for endometriosis diagnosis and management. Before 2022, the Aotearoa‐NZ health system for illness was managed by a series of 20 local district health boards (DHBs), and non‐profit Primary Health Organisations (PHOs) which were contracted to deliver a range of healthcare services [18, 19]. This regional structure was criticised as creating a ‘postcode lottery’ through the fragmentation of accessible resources and funding. This system was also viewed as insufficiently addressing health inequities for groups such as Māori and Pasifika, lacking responsiveness to consumer needs, and having significant access to care issues [20, 21]. In 2022, this system was reformed to amalgamate the DHBs and create the national, centralised Health New Zealand | Te Whatu Ora, with no formal role retained for PHOs. Important intents of the reformed system were to have both equity of access to care and of health outcomes [18]. However, it has been unclear whether current implementation plans will be sufficient to overcome the ‘postcode lottery’ element of existing Aotearoa‐NZ healthcare [19].

In parallel to these publicly funded systems, individuals have the capacity to directly access specialist services through either paying out of pocket or utilising medical insurance. While there are over five times as many surgical beds in public hospitals versus private hospitals, many specialists, particularly surgeons, work in both settings [20]. For endometriosis, this parallel private system can allow some individuals to ‘skip the queue’ rather than wait for the process of accessing a publicly‐funded gynaecologist appointment or surgical treatment. Amongst 87 endometriosis patients surveyed in Aotearoa‐NZ, 46.0% had their specialist appointments publicly funded, while 60.9% had used medical insurance, and 25.3% had paid out of pocket for some or all appointments [22]. This is higher than the Aotearoa‐NZ average where only around 15% of total health expenditure is out of pocket, and 5% is from private health insurance [20].

### 1.4 Study Purpose

Within the literature regarding endometriosis in Aotearoa‐NZ, there is a clear pattern of challenges for endometriosis patients, including long, difficult journeys to diagnosis with frequent misdiagnosis, dismissal, and traumatic experiences in care. While there is a small literature base of GP perspectives on endometriosis care from the UK, Europe and Australia, there is none to date in Aotearoa‐NZ. The purpose of this study is to identify from the GP perspective the barriers that influence endometriosis patient journeys and diagnostic delays, as well as any means through which they may be overcome. This is a vital time to identify and integrate solutions to improve endometriosis care within the Aotearoa‐NZ health system while it is already undergoing reform.

## 2 Methods and Materials

### 2.1 Ethical Approval

The design and approach to the survey study were reviewed and approved by the University of Canterbury Human Research Ethics Committee (Ref: HREC 2023/25).

### 2.2 Recruitment

This interview study was done in tandem with a survey study [23]. To recruit participants for the survey, an anonymous link and information sheet was emailed to 869 clinics, inviting GPs to participate in a survey about endometriosis care in Aotearoa‐NZ. Contact information for clinics was found on health centre websites and the information site HealthPages. Upon completion of the survey, the 185 GPs who completed the survey could register interest in conducting a follow‐up interview to discuss their views on endometriosis care in Aotearoa‐NZ. Thirteen GPs expressed interest, nine set times to conduct the interview, and nine interviews were subsequently conducted.

### 2.3 Study Design

The purpose of the study was to assess the perceptions of Aotearoa‐NZ‐based GPs on the Diagnosis and Management of Endometriosis in New Zealand guidelines, endometriosis patients, and the care provided in Aotearoa‐NZ. The interview questionnaire was partly modelled on a 2019 study by Young et al. where semi‐structured interviews were conducted with eight gynaecologists and four GPs in Australia [24]. In our semi‐structured interviews, demographic information was collected at the start of the interview, then the GPs were asked their views on: their use of the ‘Diagnosis and Management of Endometriosis in New Zealand’ guidelines, common characteristics of patients, impacts on patient lives, whether any demographic groups were more likely to have endometriosis, biases in care, and who should be responsible for the care of endometriosis patients. At the end of the interview, GPs were invited to share anything else they would like to know about the topic of endometriosis care.

### 2.4 Data Collection

When expressing interest, GPs were provided a copy of the information sheet to read and provided written consent to participate. When contacted to set a time to conduct the interview, GPs were re‐supplied with the information sheet. At the start of the interview, the GPs confirmed verbally they had received and read the information sheet, they consented to participate, and they consented to the interview being recorded. The GPs were assigned a pseudonym (GP 1–GP 9) according to the order they were interviewed, and all potentially identifying information was removed from the transcript before analysis. The GPs were provided a transcript of the interview, and had 1 week to request any alterations. The interviews were conducted by the first author by phone or over Zoom depending on the preferences of the participating GP. The question structure in Section 2.3 was followed, with the allowance for deviations to follow‐up on points raised by the interviewee. On average, the interviews were 22:58 ± 4:12 (mean ± standard deviation) minutes and seconds in duration. The interviews were conducted and transcribed by the first author. Transcription was initially done in Otter.ai, with the transcript subsequently checked manually.

### 2.5 Participant Cohort

Nine GPs set interview times and participated in this study (Table 1). One‐third of the participants were male, 5/9 had done their medical training predominantly in Aotearoa‐NZ, and the average age was 55.1 ± 12.2 years. The majority (7/9, 77.8%) had their practices in urban areas, which aligns with the majority of the population in urban areas, as 86.9% of people lived in urban areas in Aotearoa‐NZ in 2022 according to World Bank data [25].

**Table 1:** Demographics and interview features of participants.

	Sex	Age	Training location	Gynaecology CME^a^	Practice location	% Patients with endometriosis	Interview time (minutes: seconds)	‘Location’
GP 1	M	34	NZ	PGCert, Internship	Urban	3%–5%	28:06	Zoom
GP 2	F	41	NZ	No	Urban	5%–10%	23:58	Phone
GP 3	F	55	UK	Internship, General CME	Urban	3%–5%	20:37	Zoom
GP 4	M	65	NZ	PGCert	Urban	7.5%–10%	25:38	Phone
GP 5	F	63	North America	Diploma, Internship, General CME	Urban	33%	28:04	Phone
GP 6	F	59	NZ	Diploma, Family planning cert	Semi‐Rural	5%–10%	21:09	Zoom
GP 7	M	64	UK	No	Rural	2%–3%	24:00	Phone
GP 8	F	43	NZ	No	Urban	5%	21:32	Zoom
GP 9	F	72	UK	Diploma	Urban	1%	13:40	Phone

^a^ Continuing medical education.

### 2.6 Data Analysis

**Figure 1:**
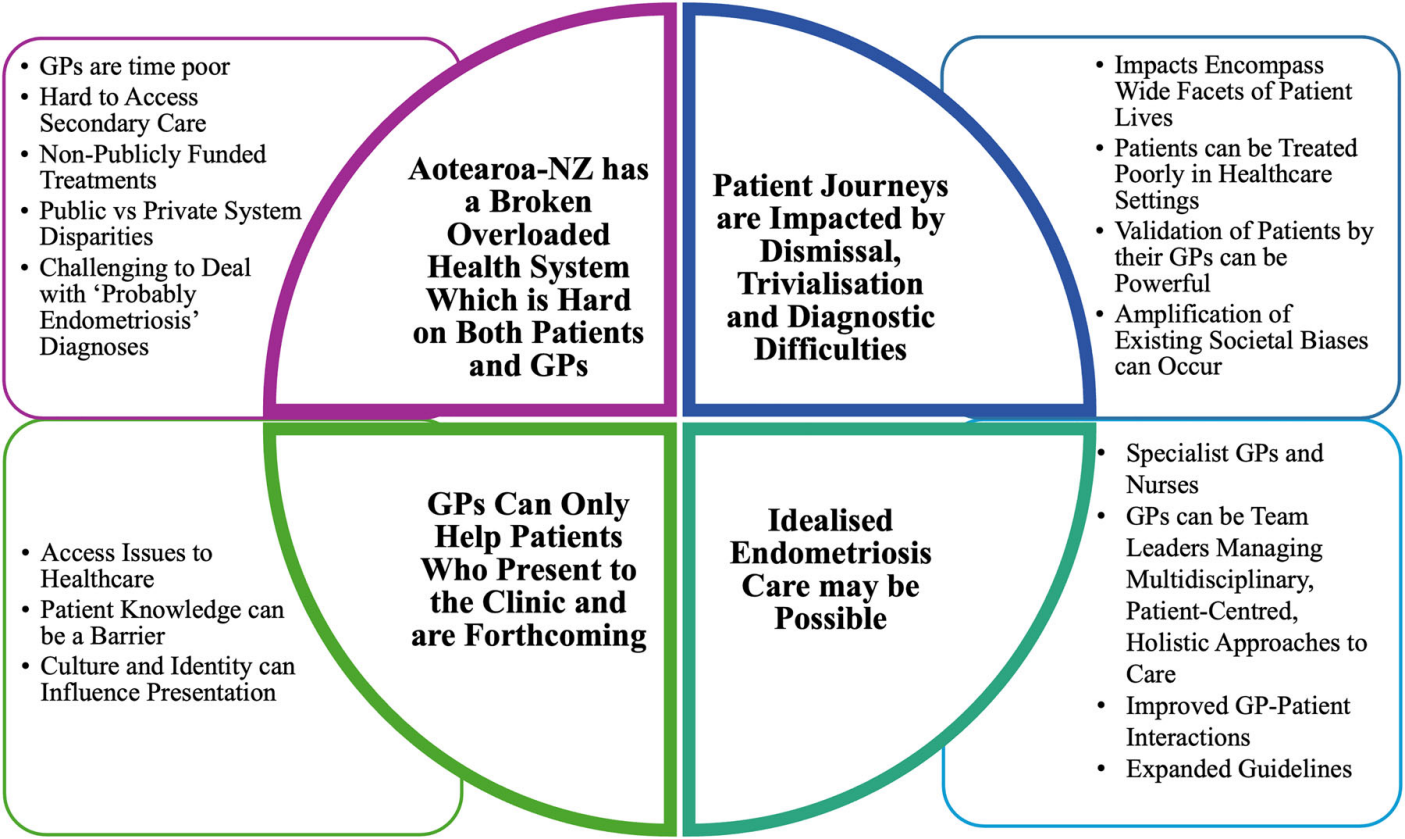
Theme map for GP interviews regarding perspectives on endometriosis care in Aotearoa‐NZ.

Interview transcripts were analysed independently by the first and second authors. Transcripts were read multiple times before thematic iterative sematic, inductive coding [26] was conducted in NVivo (version 1.6.1). The first and second authors iteratively collated their independent codes into preliminary themes and sub‐themes for comparison. After coding all nine interviews, the first and second authors then compared code books to assess the similarity in their codes and preliminary themes. Both coders identified the same key codes and collaboratively, iteratively assembled and clustered their preliminary themes into the final themes and sub‐themes included in this article (Figure 1).

Disagreements were to be handled through the incorporation of a third author to discuss the difference and reach a consensus, however, no disagreements occurred in the development of these themes. Representative quotes for each theme and subtheme were selected from the code book of one coding author, and assessed by the other coding author for their representativeness of their independently developed codes. Both coding authors identified that their codes were largely unchanged after six interviews, with no new preliminary themes emerging after this point. Coding of the further three interviews supported the consistency of these themes.

Both coding authors are cisgender women with backgrounds in qualitative analysis who have been treated for endometriosis symptoms in their lifetimes and have navigated endometriosis care in Aotearoa‐NZ. The other authors have not directly navigated endometriosis care but have backgrounds in endometriosis research, and oversaw the construction of the questionnaire and the interpretation of the themes included in this article.

## 3 Results

### 3.1 Aotearoa‐NZ Has an Overloaded Public System Which Is Hard on GPs and Patients

The perspectives of the GPs in this study indicate that the Aotearoa‐NZ public health system has more patients with more health needs than it can support. This creates systematic shortages, as practitioners must support large patient populations on short timelines, not all treatments can be subsidised, and specialist care pathways become overloaded. For GPs, this can mean they are unable to provide patients with the care, treatments, or support they would like. For patients, they cannot always utilise the diagnostic and treatment tools that may provide relief.

#### 3.1.1 Issues Within Primary Care

The GPs identified that many of the issues within primary care are not a function of the individual practitioners but of the system in which GPs are practising medicine. GPs are time‐poor in consultations, with short windows in which to assess patient symptoms and needs, as well as build the relationships that are necessary for the establishment and maintenance of trust:

Services across the spectrum are under increasing pressure… that's a barrier to effective and confident management.

(GP 7)

We can see what the best practices [are], we can see how that is. But I think that's really challenging within the timeframe to address all of those things that we need to address.

(GP 3)

Particularly in endometriosis care, where there can be menstruation‐related and symptom normalisation, this can be insufficient time for an issue to be identified, a relationship of trust to be developed, and the patient educated:

So the women don't want to talk about it because it's awkward. And practitioners haven't got time to deal with it. And there may be that underlying stuff that women's health stuff still is a bit taboo or unimportant.

(GP 5)

On top of this issue, some of the endometriosis treatments that are recommended within the Aotearoa‐NZ guidelines are unfunded. If a medication is subsidised, then there is a standard prescription cost associated with its use. However, if a medication is not subsidised, then the person prescribed the medication has to pay higher charges for use, which creates a larger access issue than would otherwise exist. For the GPs, it was frustrating to be told that a nonsubsidised medication, which may be an effective approach to treating their patients' symptoms, should be recommended to their patients, who may not be able to utilise this option because of financial barriers:

The perennial issue in New Zealand is with restricted funding.

(GP 7)

#### 3.1.2 Issues Accessing Gynaecology Services

Once menstrual‐related distress has been identified, GPs may then struggle to access further services for their patient, such as imaging, specialist consults and public treatment services (e.g., pelvic physiotherapy). While it was identified that the entire system was overloaded with insufficient resources to treat the number of patients needing them across the entire health system, GPs mentioned this issue seemed particularly pronounced in gynaecological care:

Gynaecology has always been… the service has always been very limited, but it's much worse than usual. The urologists are also in a similar situation. Other specialties we don't have so much problem with.

(GP 9)

These access issues exist throughout Aotearoa‐NZ, but are particularly pronounced in regional areas that have fewer resources at baseline, leading to a postcode lottery where access and overall treatment can vary from location to location:

Provision of care is different depending on where you live and what area… expert, ultrasound sonographers doing pelvic ultrasounds and looking specifically for stigmata of endometriosis. You know that very much has an urban [large city] lens… care for endometriosis patients will be dependent on what resources are in the area that you live.

(GP 8)

Referrals to access public gynaecologists were frequently denied to the GPs in this study. GPs highlighted that they sometimes write multiple referrals for the same patient before it may be accepted for specialist assessment, ultrasounds, or another diagnostic imaging test. The decline of these referrals was particularly frustrating as GPs were unsure what else they could do to manage these patients without secondary care support. Furthermore, each referral written used up time and resources, which felt wasted upon the decline:

You would then send another referral… which takes time and energy and effort, which would also be declined… it's just a huge delay.

(GP 1)

I keep writing referrals. Sometimes when you've referred somebody two or three times they will eventually get seen. Sometimes you're just still in the dark about whether they've got endometriosis or not.

(GP 9)

#### 3.1.3 Public and Private System Disparities

Clearly identified by many of the GPs in this study was that the public system was associated with resource shortages, long wait times, and variable quality of specialists:

I think having access to a pelvic floor physiotherapist is *so* essential and that's only available on the private system. You know, there's nobody that can access that publicly.

(GP 3)

I think as the access to public funded care becomes more difficult with longer and longer waiting times that's [using the private system] likely to increase.

(GP 7)

In the public system, you're also likely to get like a registrar or someone who might be good but… it's just variable as to who the person sees… So the quality definitely, at least in [location], is embarrassing.

(GP 1)

It was also identified that, for an individual patient, all of these issues could be circumvented by having the financial capacity to utilise the private system. Patients who could pay out of pocket or utilise medical insurance (which is opt‐in within the Aotearoa‐NZ health system) could fast‐track specialist appointments and imaging, have shorter overall wait times, and select the specialist they wanted. It was generally accepted that these patients would potentially have better outcomes than the patients unable to escape the slow and under‐resourced public healthcare system:

[Private healthcare] certainly is an easier pathway for them to go once endometriosis is flagged or known, and they kind of get to the point where they want a referral, it's just a faster, easier process with fewer of those frustrations.

(GP 2)

There aren't the same capacity issues if you can afford to go privately.

(GP 9)

#### 3.1.4 Challenging ‘Probably Endometriosis’ Diagnoses

In light of these issues, GPs are frequently put into the position of managing patients with menstrual‐related distress purely within primary care. For these patients, they could only offer a ‘*probably endo*’ diagnosis in the absence of the possibility of radiological or surgical confirmation. GPs in this study described this status as quite an ambiguous position to treat patients from, as they then had to focus purely on symptom control:

I know “*probably endo*” means they've got abdominal pain or pelvic pain. Nobody has figured out what is going on, so they lump them in to “*you've probably got endo*,” so that is disastrous for equity in healthcare, really.

(GP 4)

### 3.2 Patient Journeys Are Impacted by Dismissal, Trivialisation and Diagnostic Difficulties

#### 3.2.1 Impacts on Endometriosis Patients

The GPs in this study identified that endometriosis could impact any and all aspects of a patient's life. The result of this is that patients could be prevented from being able to live the way they want:

[Patients] have a certain expectation about living a certain way in society… you should be doing everything… Like everybody else. And if you don't want to behave like you're an outsider – and that's a different kind of suffering that those women experience in addition to the physical pain of the endometriosis condition.

(GP 5)

**Figure 2:**
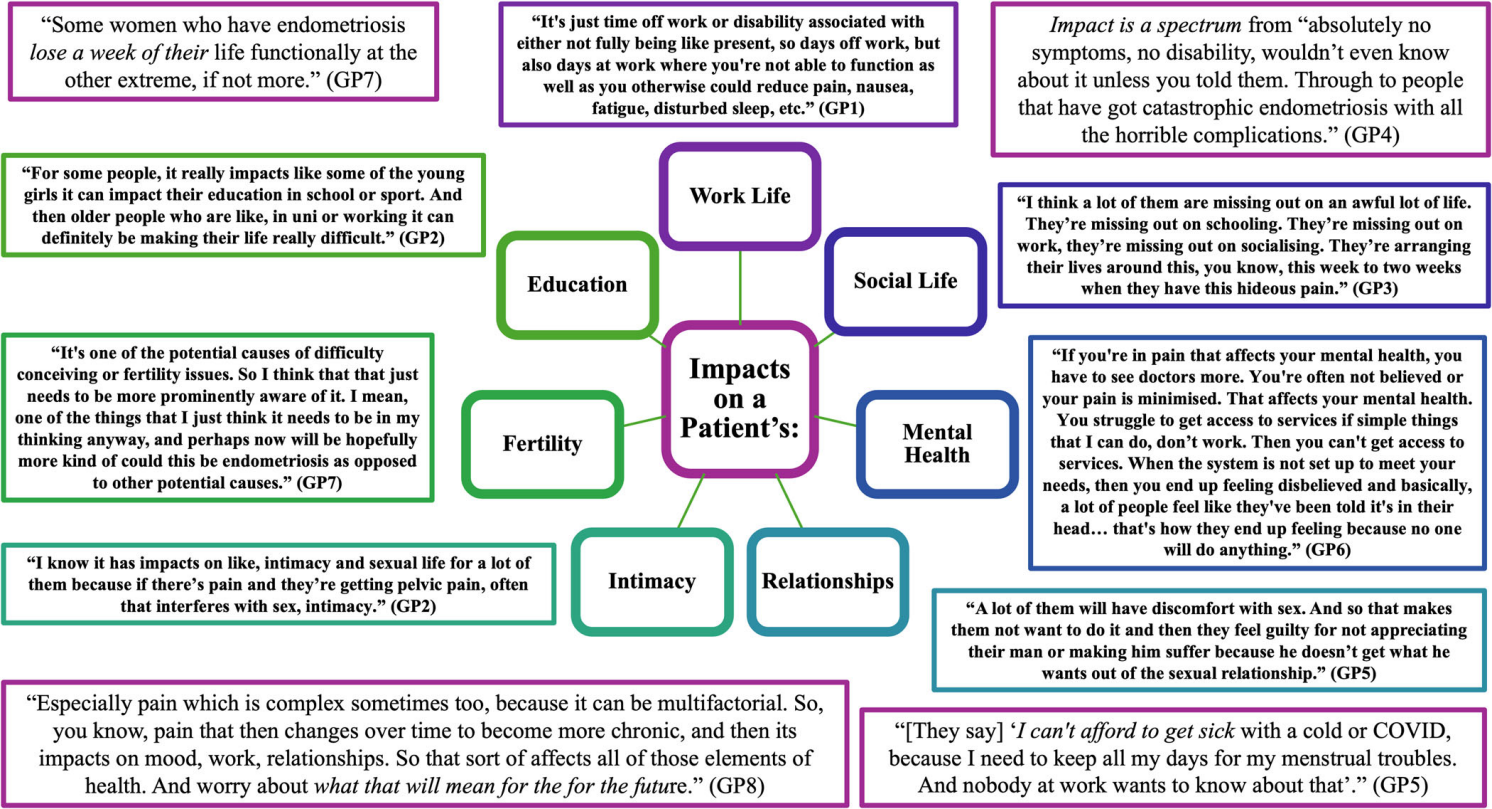
Summary of quotes associated with the impacts of endometriosis on patient lives.

GPs identified holistic, all‐encompassing impacts of endometriosis on areas as diverse as their work, education, and social lives, relationships and intimacy, infertility and mental health (Figure 2).

#### 3.2.2 Poor Treatment and the Power of Validation

The GPs identified that, while not within their personal practice, they were aware endometriosis patients could be treated poorly within healthcare settings – facing dismissal, trivialisation and normalisation of their symptoms. For one GP, those experiences were personal:

My perceptions are a bit coloured by the fact that I had endometriosis… [I] saw how badly women presenting with period pain were treated, and I thought, I don't want to go there because I don't want to be judged.

(GP 6)

Patients facing dismissal can find themselves invalidated, judged, not listened to, not believed, and having their pain minimised. GPs indicated their patients had previously been told by healthcare professionals directly or indirectly to ‘toughen up’ (GP 3) and to ‘live with [heavy and painful periods]’ (GP 4). These experiences for patients could tie in with the impacts on patient mental health (Figure 2), creating a ‘hidden morbidity’ (GP 7) of endometriosis‐associated mental health impacts due to dismissal in healthcare spaces (Table 2).

**Table 2:** Quotes associated with the subthemes of the theme ‘Patient Journeys are Impacted by Dismissal, Trivialisation and Diagnostic Difficulties’.

Subtheme	Quote	GP
Poor treatment and power of validation	‘I would say 75%‐80% of [my patients] have not been helped [in healthcare], and I would say at least half of them have actually been ‘harmed’ in the sense of the misinformation and the sometimes lack of empathy for what they are actually going through. It's been minimalised… side‐lined, and to be frank, all the bullshit diagnoses, misinformation [is really harmful].’	GP 4
	‘I think it's not just the illness, it's the impact of the illness on their mental health as in like they're experiencing pain and having time off and not living their life in the ways that they want to… a lot of women feel kind of dismissed or not heard through their healthcare journey.’	GP 2
	‘So you almost need to tell people to be really grounded when they go into the consults with gynae. Otherwise, they'll just dismiss them… some surgeons will be like ‘yes, tears… be free’. Other people will just be like ‘they're making this pain up’… In general practice, actually, if you validate people and tell them that they're not crazy and the pain isn't in their head… I think validating [] is really helpful.’	GP 1
	‘I just don't refer to them [a particular specialist] anymore because I know they're going to get treatment that is very medically‐physically‐based without anything else and it's quite dangerous, as endometriosis is a whole‐person disease.’	GP 6
	‘Very often at the end of the consultation they say “thank you for listening. I feel like you took respect for what I said. I don't often get heard. I don't know why nobody's explained this to me before or offered me this particular course of action.”’	GP 5
Amplification of existing equity issues	‘The usual inequities in healthcare are very likely to make the experience of endometriosis worse for the patient.’	GP 4
	‘I think there's significant bias against Māori, Pasifika, Asian, refugee populations because either they don't come [to GP appointments], or they don't articulate the issue, or they don't know how to articulate the issue, or they don't know how to ask for help.’	GP 3
	‘I think historically [it has not] been recognised early enough in presentation as a possible diagnosis. Then, where there are other access to healthcare issues or health literacy issues – those will be magnified by that.’	GP 7

These issues were not only identified to be present within primary care. GPs indicated that specialists could also be a source of dismissal and poor treatment for endometriosis patients. These attitudes of specialists could cause GPs to warn their patients to present with ‘reasonableness’ (GP 1), i.e., not presenting with emotion or tears, in front of specialists to avoid this dismissal. When aware there were particular specialists they would prefer to have their patients see, the GPs may also selectively refer their patients to certain specialists they have faith will listen to their patients and validate their experiences (Table 2).

The inverse of the experiences of patients facing dismissal was the experience of patients when their GPs validated their experiences. There was a sense that the validation was powerful for patients and enabled the creation of a more effective therapeutic alliance to tackle the symptoms and impacts associated with endometriosis. Some patients may see a series of GPs who treat them poorly and dismiss them before then meeting a GP who validates their symptoms and experiences, which can be quite a poignant moment for patients (Table 2).

#### 3.2.3 Amplification of Existing Equity Issues

Patients with any existing access issues, such as cultural barriers to discussing menstruation, financial barriers to paying for or travelling to GP visits, or mental health issues (which may be exacerbated by endometriosis), can have these amplified within endometriosis care. For example, endometriosis management involves numerous follow‐up appointments with GPs to be effective. Patients who have financial issues are likely to struggle with this need for repeated appointments, as each can require an out‐of‐pocket cost for the patient. Barriers can arise through the continual need to pay for prescriptions and appointments. This can mean patients will require leave from work and transportation to appointments, which may make patients with existing equity issues more likely to stop seeking this medical support. These types of financial barriers are part of the trend identified of existing equity issues such as racial biases, cultural taboos, and low health literacy being amplified within endometriosis care (Table 2).

### 3.3 GPs Can Only Help Patients Who Present to the Clinic and Are Forthcoming

GPs in this study identified that some patients struggle to be open and forthcoming about menstrual‐related distress and endometriosis symptoms. These issues include the knowledge patients do or do not have while first experiencing endometriosis symptoms and the cultural and social factors that influence their view of these symptoms.

#### 3.3.1 Patient Knowledge Pre‐Diagnosis

When experiencing endometriosis symptoms, patients need to have an awareness that their symptoms are a cause for concern to know they should seek medical attention. The barriers GPs described which could prevent patients from identifying their symptoms warrant medical attention include having low levels of health literacy. This could manifest as a belief that endometriosis symptoms are ‘normal’ and therefore do not need to be assessed.

I find that people with lower health literacy are less likely to present, they're less likely to actually think that it's a problem because it's very much like ‘well it's a women's lot. You have to, you know, deal with it’.

(GP 3)

So if people assume that their severe debilitating pain is normal, and they don't bring it to us as a problem, then they won't get diagnosed or managed.

(GP 1)

#### 3.3.2 Culture and Identities

zAdditional barriers to patients realising their symptoms are pathological, visiting a GP, and feeling comfortable sharing about their symptoms could result from characteristics of their backgrounds, particularly cultural or ethnic background. These concerns and barriers could be particularly clustered around the willingness and comfort discussing menstrual‐related and other endometriotic symptoms with GPs. The GPs in this study did not generally think certain ethnicities or groups would have a biologically higher incidence of endometriosis. However, in their practices, they saw fewer endometriosis patients who identified as Māori, Pasifika, Asian, or another ethnic minority (i.e., not of European descent).

This discrepancy could result from these patients experiencing a cultural taboo or reticence to discuss reproductive health, or, because of normalisation of pain within family groups. These patients can have much greater difficulties in feeling willing to present to a GP clinic, being open to discussing reproductive health, and finally, feeling comfortable or able to return to GPs when primary treatment approaches may not work right away (Table 3).

**Table 3:** Quotes associated with the subthemes of the theme ‘GPs Can Only Help Patients Who Present to the Clinic and are Forthcoming’.

Subtheme	Quote	GP
Culture and identities	‘My main concern is that like, we don't know what we're missing in terms of Māori and Pacific because I feel like, especially for Pasifika, it is kind of just this like grin and bear it.’	GP 1
	‘I think culturally Māori often just put up with a lot of women's health conditions that when I hear these stories that sound to have significant impact on their lives and work. Whether that is due to intergenerational experience… unfortunately they just solider [on] and put up with things that maybe other cultures or other demographics have not or would not.’	GP 8
	‘I have a relatively high proportion of Asian population and also a refugee population… they don't present and if they do present, it's often very hard, especially if there's a male interpreter, to sort of talk about periods.’	GP 3
	‘So I don't see very many Pasifika or Māori women. And the few that I can think of that I think have endometriosis and I'm trying to treat, particularly with really heavy bleeding, they don't tend to come back and they don't tend to persevere with treatment.’	GP 5
Characteristics of presenting patients	‘There is a subset of people who come in who are very empowered because they've gone: ‘I know I've got endometriosis. I know I want you to fix this. You do this for me.’ and that's great. But they're few and far between. Most people are sort of quite exhausted, quite depleted by it… they don't feel they can stand up to endometriosis’	GP 3
	‘I think the overall thing for me that the characteristic [shared by patients] is fear. So there is a real fear of endometriosis because young people think that endometriosis… makes you completely disabled ‐ you can't work, you can't study, you have to be on a benefit… your life will be a misery. And that's a really strong fear in them with that. And unless you actually address that at the very beginning, you fight a losing battle.’	GP 4
	‘The young women, I think they feel really discouraged. They feel like they're suffering and that's been going on for a long time and they're tired and uncomfortable and they feel really badly about themselves.’	GP 5
	‘There's a lot of frustration with their whole experience with their journey through the healthcare system.’	GP 2
Information from GPs during patient journeys	‘Even if you do [have consistency of care and] someone says, ‘I think this is endometriosis’, it's almost just like in passing, or just in the last 5 min of a consult… people don't really have kind of a sit‐down conversation about what this means and what it implies… when someone's diagnosed with diabetes, then they get a six‐week programme of diabetes education… with endo, it's just kind of like a throwaway statement.’	GP 2
	‘Usually by the time I'm seeing them, their head is full of crap that their mothers or their auntie or their grandmother told them, or another doctor or nurses told them, or they read on Google. And sometimes all of those sources are wonderful and [the] patient has a good experience with that information. [But] I would say at least half the time, that is a bad experience for them and the information they get is frankly misinformation for their context.’	GP 4

#### 3.3.3 Characteristics of Presenting Patients

The GPs generally found there was a common patient profile amongst those that did present to GP clinics to seek care for endometriotic symptoms. The presenting patients were commonly described as exhausted, fearful, concerned and frustrated. Patients expressed these emotions both directly in response to the symptoms being experienced as well as the difficulty of their diagnostic and management journeys (Table 3).

#### 3.3.4 Information From GPs During Patient Journeys

The information patients had previously received about endometriosis could also pose a challenge to practitioners. This would be when this information was viewed by practitioners to be poor quality, or to be misinformation, e.g., that all endometriosis patients will be infertile. This is linked with the perception that it can be difficult for patients to consistently receive high‐quality, accurate information about endometriosis, including during the diagnostic period (Table 3).

### 3.4 Idealised Endometriosis Care

There was a general sentiment that endometriosis care has improved over the last few decades as awareness amongst the general public and medical practitioners has improved:

I think it's better than it was 10–15 years ago, so I think it's improving!

(GP 3)

Despite these improvements, the GPs in this study had ideas for how the care provided to patients could be further streamlined and improved. This included incorporating specialist GPs and nurses, placing GPs into team leader roles and improving the standard of GP‐patient interactions.

#### 3.4.1 Specialist GPs and Nurses

GPs in this study felt that often the best GPs for supporting endometriosis patients were those with a particular interest and training in women's, female reproductive and menstrual health topics. GPs identified remaining up to date with these topics was important. However, there is an immense annual volume of continuing medical education (CME) across a wide range of topics, making keeping up challenging:

There's so much CME that you could do all the time.

(GP 5)

The problem that we have as GPs is that we have so many guidelines, you know, there's guidelines for everything out there.

(GP 3)

Therefore, it was recommended that there should be GPs or nurses within clinics who are dedicated to keeping up to date with this gynaecological CME. This would create a workflow within clinics where menstrual‐related discomfort can be identified by all medical practitioners within the clinic, with patients then streamlined to the practitioners with an interest and expertise in those conditions:

It may be in those sorts of practices, they would have somebody who has a particular interest and expertise in women's health for example ‐ and that might not necessarily be a GP ‐ it might be a nurse practitioner, or a specialist nurse with particular skills in that area.

(GP 7)

#### 3.4.2 GPs as Team Leaders

Another workflow identified as potentially beneficial to patients and GPs was reconfiguring the role of GPs in endometriosis care. The GPs of this study identified that endometriosis care is most effective when it is multidisciplinary, holistic and patient‐centred:

Endometriosis is a whole person disease… it's a whole lot of things ‐ you know, social, mental health, physical health… relationship health. It's a whole big thing.

(GP 6)

I think that the people that get the most benefit are those that have input from gynaecologists… pelvic pain specialists… pelvic physiotherapists, people who specialise in pain management… in the psychological aspect, and then you've got the impact of the dieticians. So, it's such a holistic thing!

(GP 3)

In this proposed setup, GPs are the team leader or manager of their patients' endometriosis care. The GP will take the primary role of caring for and supporting the patient; designate the multidisciplinary team that should be treating the patient (such as radiology, pelvic physiotherapy, psychotherapy, dietician); and track progress of this multidisciplinary approach:

The clinician is responsible for building a therapeutic relationship with the patient and listening, hearing what's most important for them, hearing about their fears and expectations and hopes, and then… shared decision‐making as to how to reach that outcome. That use of allied health: so psychologists, physiotherapists, dietician, health coach ‐ all of those people are responsible for the care… then whether that involves secondary care, or a gynaecologist with an interest, then that person would join the team.

(GP 8)

However, this approach is likely impossible without first addressing the access issues to these services which the GPs of this study identified as making endometriosis care particularly challenging.

#### 3.4.3 GP–Patient Interactions

Fundamental to improving the overall approach to endometriosis care in Aotearoa‐NZ are strong, positive, beneficial relationships between GPs and their endometriosis patients built upon good rapport and trust. Validation and active listening from GPs towards their patients were identified as having powerful implications for patients. To ensure patients continue to present to GPs for iterative improvements to a management plan tailored to the patient, patients need to be centred and actively involved in their care.

#### 3.4.4 Additions and Alterations to the Endometriosis Guidelines

**Figure 3:**
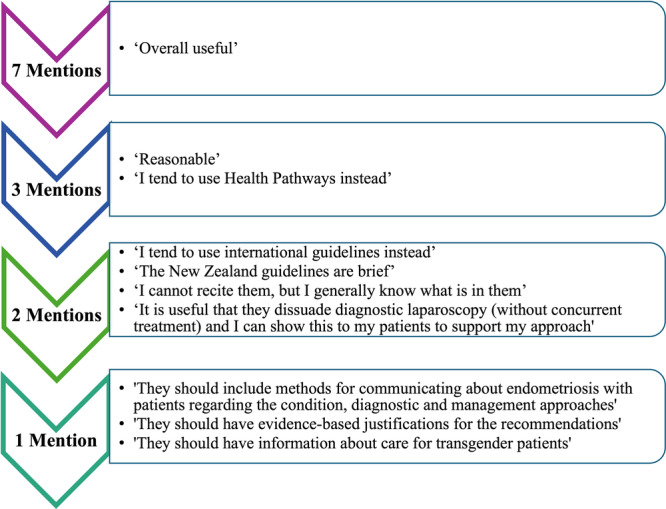
Sentiments expressed regarding the current ‘Diagnosis and Management of Endometriosis in New Zealand’ guidelines.

Regarding the guidelines, the GPs interviews generally had positive perspectives, and several suggestions for future iterations (Figure 3). It was recommended that the guideline should be a clinical, centralised guideline that GPs can be sure is up‐to‐date and evidence‐based. In particular, GPs highlighted that there should be strong roll‐out communications to encourage GPs to read and engage with the updated guidelines. Regarding the potential of guidelines, GP 4 highlighted:

I do not think there are a bunch of anti‐endometriosis practitioners out there. They are not born that way; they get that way by experience and the information that is stuck under their noses. So if we change that information, I am hopeful the attitudes will change.

## 4 Discussion

### 4.1 The Barrier of Diagnostic Difficulty

The GPs within this cohort described seeking to treat endometriosis patients within an overloaded health system where referrals would be repeatedly denied, preventing them from accessing specialist care and imaging for their patients. This situation leaves GPs in the scenario of utilising a ‘*probably endometriosis*’ diagnosis for their patients, which comes with its own challenges. There were indications that endometriosis was not being considered a priority for imaging referrals, and that in the overloaded system in some regions, only imaging for gynaecological cancer screening was occurring. This aligns with a study from the Netherlands where there was a sense of low urgency for endometriosis diagnosis contributing to diagnostic delay [27].

Diagnostic imaging for endometriosis can be challenging. Patients can have false‐negative or inconclusive laparoscopies before later surgical confirmation of the disease [28]. Endometriosis, particularly of the superficial type, can also be missed during transvaginal ultrasounds [29]. The capacity to identify endometriosis through imaging modalities is rapidly improving, particularly for ovarian and deep‐infiltrating types. To take advantage of these advancements requires investment into training, and then accessibility for patients to practitioners who understand the application and interpretation of specialist techniques [30, 31].

This working or suspected diagnosis can bring relief to patients as it marks that their GP has listened to them, believed them, and legitimised their patient status [3]. For GPs, however, it can remain unclear the precise pathway they should utilise with these patients. It was difficult for GPs to know whether applying this label to the patient and progressing with management on the assumption of endometriosis would benefit their patient's health in the long term. This challenge has also been identified in an Australian GP cohort, where GPs similarly highlighted issues accessing public specialist services for their patients for guidance on either accessing a diagnosis or treating in the absence of lesion confirmation [11]. It is consistent in Australian GP cohorts, this current GP cohort, and Aotearoa‐NZ‐based patient cohorts that poor public access to specialist care creates a reliance upon patients utilising private gynaecology services through out‐of‐pocket payments and medical insurance [11, 32].

It is well established in qualitative studies with patients that interactions with medical practitioners can either be facilitators or barriers to effective endometriosis diagnosis and treatment [32, 33, 34, 35]. For example, diagnosis can be facilitated by patient symptoms being acknowledged by a practitioner with an up‐to‐date knowledge base, while a lack of awareness, and symptom normalisation by practitioners can present a barrier [33, 35]. The pain associated with endometriosis has been implicated as a source of miscommunication between patients and their GPs. Both healthcare practitioners and patients can incorrectly accept menstrual‐related pain as normal [36]. Patients can struggle to communicate the nature of their pain through existing quantitative methods (such as pain scales) [37], which are commonly used in primary care.

### 4.2 The Barrier of Limited Time

The challenge of assessing the complexity associated with endometriosis‐related symptomology in short consultation sessions was raised in this GP cohort. In Australian studies, it has been similarly identified that there can be missed opportunities for GPs to identify endometriosis when there is not sufficient time available to identify warning signs [11, 12]. Furthermore, patients who feel rushed in consultations can feel dismissed and that their pain has been normalised [37]. Endometriosis management is complex, and not all management strategies will be effective immediately. This creates a requirement for repeated GP visits to assess the effectiveness of treatments over time. The necessity for ongoing, iterative assessment means GPs need to be able to dedicate more time, not less, to endometriosis patients. The present systems in Aotearoa‐NZ and Australia do not allow for this [11].

Within consultations, GPs can face additional challenges as a result of language barriers, the perception of symptoms as taboo, and the difficulty in discerning qualitative descriptors of endometriosis‐related pain from other pain pathologies [11, 12, 37]. In an Aotearoa‐NZ cohort of Māori and Pasifika patients, it was highlighted that the shame of discussing endometriosis‐related symptoms could be exacerbated when needing to communicate these topics within spaces that lacked cultural safety and so felt inhospitable [38]. These patients also experienced high rates of weight‐based discrimination, drug‐seeking accusations and racism when trying to access endometriosis care [38]. Similarly, LGBTQIA+ endometriosis patients could struggle to communicate their needs and desires within medical spaces. Experiences of homophobia and transphobia when navigating care could cause them to choose to remain ‘closeted’ to their medical practitioner, causing their practitioners to be unaware of priorities within their treatment (such as to not utilise medications with estrogenic compounds) [39].

In an Australian study of patients and healthcare practitioners, both groups independently identified the value of validation of endometriosis patients in the therapeutic relationship. This allows for shared decision‐making, with utilisation of both the patient's and the practitioner's expertise [12]. To build this effective therapeutic relationship, which is highlighted as an important pathway for better endometriosis management [11, 12, 40], GPs in this study indicated they need the capacity to spend more time with their patients in consultations. Practicing patient‐centred care and validating patient experiences is likely to be well accepted by patients. In a 13‐country European cohort assessing patient‐centredness of endometriosis care, the top‐ranked dimension for ensuring patient‐centredness by endometriosis patients was ‘respect for patients' values, preferences and expressed needs’ [41]. To accomplish this, it has been suggested that GPs may benefit from an approach that incorporates an understanding that patients may have had prolonged periods of symptoms before their appointment. Furthermore, GPs should encourage patients to maintain symptom diaries, avoid assumptions of patient therapeutic priorities (such as emphasising fertility), and establish that the therapeutic relationship will be collaborative and ongoing [40].

### 4.3 The Barrier of a System With Limited Resources for Endometriosis Care

Further initiatives proposed for reducing the delay to diagnosis include [12, 36, 42, 43]:

- Education and skill‐building around endometriosis for patients, the general public and practitioners
- Updating diagnostic guidelines with attached dissemination and implementation plans
- Developing standardised checklists for symptoms
- Establishment of multidisciplinary care teams
- Allocating appropriate time and resources for effective clinical assessment, counselling and patient education
- Advocating with health ministries to improve attention to endometriosis
- Securing funding to address practitioner knowledge gaps

#### 4.3.1 Education and Upskilling

The GPs of this study indicated that having specialist GPs or nurses that remain up to date with endometriosis best practice would be an effective means of ensuring patients can be funnelled to a practitioner who is well placed to support them. This would also allow funding for training and upskilling to be funnelled towards a specific, smaller cohort of practitioners. Further research should be conducted with nurses to determine their perspectives on whether specialist nursing for endometriosis roles and training would be of interest, and nurses should then be central in the design of any programmes and schemes. This potential curriculum should be developed with the support of organisations such as the Royal Australia and New Zealand College of Obstetricians and Gynaecologists, the Royal New Zealand College of General Practitioners, the Nursing Council of New Zealand and patient organisations to ensure any training is consistent, needs‐based, fit‐for‐purpose, accredited, feasible and implementable.

#### 4.3.2 Updating Diagnostic Guidelines

In 2023, the New Zealand Government released its Women's Health Strategy, which sought to set long‐term priorities to address women's health issues for the next ten years. The goals of this strategy were to create a health system that provided equitable health outcomes between men and women, and between all groups of women [44]. Endometriosis was highlighted in this health strategy as an example of concern, with a lack of both specialist treatment availability, and management guidance for practitioners, resulting in an unfair lack of provision of effective healthcare for patients [44]. The findings in the strategy document highlight that to address the health inequities imposed by endometriosis, the supports for medical practitioners in the provision of care must be addressed and improved.

The ‘Diagnosis and Management of Endometriosis in New Zealand’ nonclinical guideline [45] was released in 2020, but in the tandem study that surveyed 185 GPs, only 35.1% reported that they had read the document [23]. In this survey study, 48.4% indicated they did not or only somewhat felt they had sufficient knowledge about endometriosis for their routine practice [23]. GPs in this interview cohort made several suggestions for how these guidelines could be updated and expanded, with an emphasis placed on ensuring there is an effective roll‐out of said updated guidelines to ensure uptake by a wide range of GPs. Suggestions in the literature include improved pain communication tools [37], diagnostic decision flowcharts that incorporate menstrual health and endometriosis screening questions [11, 46], and incorporating endometriosis into the differential diagnosis guidelines for related conditions (such as irritable bowel syndrome guidelines) [46].

#### 4.3.3 Multidisciplinary Teams

Improvements to healthcare provision have been suggested as part of a 3D model by Lukac et al., with targeted work with both existing physicians and medical students, while also creating specialised roles for practitioners such as nurses [36]. The proposition that improved understanding must be targeted at both presently practicing GPs and trainees is also reflected in prior studies with GPs and gynaecologists [11, 46]. Multidisciplinary teams, particularly at accredited endometriosis expert centres, are frequently lauded as an approach to improve the provision of endometriosis care [47, 48, 49]. In an Italian study of 722 endometriosis patients, 45% considered a specialised endometriosis centre to be a necessity [50]. Multidisciplinary care teams can include members such as physiotherapists [51], gastroenterologists, urologists, radiologists [42], gynaecologists, psychologists and pain specialists [42, 51]. In this study, GPs indicated that their role in endometriosis care should be as the coordinators of holistic, multidisciplinary approaches to care. However, they stressed that this scenario was only viable within a system where there is sufficient public access to this style of multidisciplinary care. Presently, to access these practitioners, there can be substantial lag periods with repeated referral processes or substantial financial barriers that re‐enforce a public–private healthcare divide.

#### 4.3.4 Resourcing for Longer and More Effective Consultations

With or without the structure of specialist endometriosis GPs or nurses, the GPs in this study highlighted that there still needs to be the capacity for provision of consultations with sufficient time for relationship‐building, priority setting and establishment of holistic care plans. This may be achievable through targeted funding for longer initial consultations for complex, chronic conditions such as endometriosis. Another avenue to improve patient support and education would be a free, nationwide telehealth service where patients can be educated, validated, directed to accurate resources and supported in accessing pathways to effective management.

Ideally, there would be publicly funded specialised endometriosis and persistent pelvic pain clinics throughout the country. These could then be complemented by mobile clinics for hard‐to‐access populations, could ensure access to timely support, diagnosis and management. All of these solutions would need to have effective grounding in the provision of culturally safe care, and antibias training to avoid replication of the systems and attitudes that have been hurtful to patients with minoritised backgrounds in Aotearoa‐NZ to date [38, 39]. Investment in the design and development of these kinds of programs would contribute to the improved provision of equitable care for all endometriosis patients highlighted as a priority in the Women's Health Strategy [44]. This could also contribute to the goals of equitable access to care and health outcomes of the 2022 healthcare reforms [18]. To align with key goals of equitable healthcare, key solutions should be designed from the outset with the intent of being publicly available and readily accessible without replication of factors that contribute to patient disenfranchisement.

### 4.4 Study Limitations and Strengths

This study has several limitations worth noting. First, as a result of the qualitative nature of the study, the overall sample size is small. However, it was noted by both authors who thematically analysed the transcripts (K.E. and A.M) that data saturation had been reached before the ninth interview. The overall interview lengths were short (13–28 min) as a result of the very busy schedules of the target audience, and more insights would be able to be gained through longer interviews with a more extensive interview guide. Thirdly, the results are likely biased towards GPs who have a greater interest in endometriosis and likely a greater knowledge about endometriosis, since participating GPs first chose to participate in a survey about endometriosis, then signed up to further participate in an interview about endometriosis.

This bias may be reflected in the empathy of the GP descriptions about endometriosis impacts, and the finding of the value of patient validation within this study. These findings are not necessarily represented in the samples of some prior studies with endometriosis medical practitioners [24, 52] or in many of the experiences of endometriosis patients in Aotearoa‐NZ studies [22, 32]. This indicates there may be a different trend in how the GPs of this study, with a greater interest in endometriosis, act and treat patients (and therefore perceive positive re‐enforcement for patients as beneficial) versus the overall population of GPs in Aotearoa‐NZ.

Further studies of Aotearoa‐NZ GP perspectives should assess whether the same or similar themes appear in a cohort devoid of this potential bias towards interest in endometriosis care and motivation to see change. These studies could be conducted by utilising a recruitment strategy of running focus groups within GP trainee classes or clinics, which has been previously attempted or successful [27, 46], or by random selection of GPs to yield samples that are less biased through not self‐selecting to participate.

A key strength of this study is that the sample includes participants with diverse backgrounds with male and female GPs of a range of ages, who trained in both Aotearoa‐NZ and overseas, with and without further gynaecology training, and who practice in both rural and urban areas. This study takes place in a nation with a public health system with a parallel private health system accessible through paying out of pocket or through utilisation of health insurance. The setting of this study means that the findings and suggestions for improvements to care are likely to be relevant to nations with similar systems, such as Australia, Canada and the UK. The solutions that are proposed within this article, such as endometriosis specialist nurses, have the potential to be similarly feasible in these other health system settings. This study takes place at an important time for Aotearoa‐NZ, as this is during a period of health reforms, and under the Women's Health Strategy of 2023‐2033. Aotearoa‐NZ has the capacity to be an important location for the roll‐out of pilot programs for improving endometriosis care, which should have highly transferrable results to other settings.

## 5 Conclusions

Nine GPs self‐selected to participate in semi‐structured interviews to share their perspectives on endometriosis care in Aotearoa‐NZ. The GPs indicated frustration with supporting patients within the Aotearoa‐NZ public health system, as they often could not access publicly funded imaging, specialist appointments, or management strategies for their patients. They highlighted that patient journeys are challenging, with impacts on all facets of patient lives possible from endometriosis. GPs identified that patients appeared to benefit from receiving validation of their experiences from their GP. Endometriosis care was highlighted as being impacted by existing challenges and biases, such as patient health literacy, taboos and shame in disclosure of endometriosis‐related symptoms, and language and cultural barriers. Finally, it was posited that idealised endometriosis care may be possible through having a system with specialist GPs or nurses, multidisciplinary, holistic endometriosis teams and expanded guidelines; however, this idealised care cannot be achieved without substantial improvements to overall healthcare access in Aotearoa‐NZ.

## Author Contributions

**Katherine Ellis:** conceptualisation, investigation, writing – original draft, visualisation, formal analysis. **Alina Meador:** conceptualisation, investigation, writing – review and editing, formal analysis. **Anna Ponnampalam:** supervision, writing – review and editing, conceptualisation. **Rachael Wood:** supervision, writing – review and editing, conceptualisation.

## Ethics Statement

The design and approach to the survey study were reviewed and approved by the University of Canterbury Human Research Ethics Committee (Ref: HREC 2023/25). When expressing interest, GPs were provided a copy of the information sheet to read and provided written consent to participate. When contacted to set a time to conduct the interview, GPs were re‐supplied with the information sheet. At the start of the interview, the GPs confirmed verbally that they had received and read the information sheet, that they consented to participate, and that they consented to the interview being recorded.

## Conflicts of Interest

Katherine Ellis is the co‐ordinator of research project Endometriosis New Zealand.

## Supporting information

Supplementary Material ‐ Interview Guide.

## Data Availability

The data that support the findings of this study are available upon reasonable request from the corresponding author. The data are not publicly available due to privacy or ethical restrictions. The interview guide is available upon request from the corresponding author.
