# Supplementary material for: ‘The Difficulty of Diagnosis Compromises Patient Care for People With Endometriosis’: Interview Study With Aotearoa New Zealand General Practitioners
Source: Health Expect. 2025 Aug 19;28(4):e70390. doi: 10.1111/hex.70390 (PMC12363337; doi:10.1111/hex.70390)
Supplement: Supplementary file 1 — Supplementary Material ‐ Interview Guide. [file HEX-28-e70390-s001.docx]

**Semi-structured interviews:**

Kia ora [name]. My name is [interviewer’s name] and I am conducting this interview today to better understand the current care provided to endometriosis patients in New Zealand.

Thank you for agreeing to take the time to participate in this interview.

Before we start, can you please confirm you have received and read the information sheet about this interview, and that you consent to participating. (Wait then ‘thank you’)

Our discussion will be recorded so that I can refer back to our conversation at a later date. I will provide you with the transcript and you will have a week to review it and make any alterations to it. Can you please confirm you are happy with our discussion being recorded? (Wait then ‘thank you’)

I would like to reiterate our conversation will remain anonymous and anything you share with me today will not be able to be traced back to you.

**As you know, we are interested in the perspectives of general practitioners of endometriosis care in New Zealand.**

**First I would just like to confirm your demographics, and we would also like to reiterate that no information you have shared will be able to be identified as you:**

Do you identify as male, female, or gender diverse?

Just to start off with, can you please share your age?

Where did you train to be a GP?

Have you completed further gynaecology training?

Do you work in an urban, semi-rural, or rural area?

And finally, what percentage of your workload do you think is made up of endometriosis patients?

Thank you. Our chat today will be divided roughly into 2 main areas, first your opinion on the current guidelines, and then about the care of endometriosis patients in general.

Now, starting with the guidelines, are you aware of the “Diagnosis and Management of Endometriosis in New Zealand” guidelines? (Wait)

What are your perceptions of the guidelines? (Wait)

Do you think the guidelines are useful? (Wait)

In what ways could the guidelines be changed so that they are of more value to GPs? (Wait)

**Now, in this section, we want to understand more about your experiences with patients in your practice.**

What do you think are the most common endometriosis symptoms?

What is your view of patients who experience this condition – do they share some common characteristics?

What impact do you think endometriosis has on a patient’s work life?... social life?... intimate relationships?... fertility?... mental health?

From your experience, do you think there are some demographic groups in New Zealand that are more likely to suffer from endometriosis than others? If so, which ones?

Can you please share your views and perceptions on the overall quality of the care provided to endometriosis patients in New Zealand?

Do you think there are any biases in the care of endometriosis patients in New Zealand?

(**If yes**) What biases do you think affect the care of endometriosis patients?

Who should be responsible for the care of endometriosis patients?... Do you think GPs are equipped to provide this care?

**At the end of the interview:**

Is there anything else you would like to share?

Thank you very much for sharing your views and experiences, it is very much appreciated.
